# A theory-driven candidate annotation architecture for collective regulation under stress in human-centered computational psychiatry: early modern Iberia as a worked coding demonstration

**DOI:** 10.3389/fpsyt.2026.1859236

**Published:** 2026-05-26

**Authors:** Eik Niederlohmann

**Affiliations:** Department of Psychosomatic Medicine and Psychotherapy, Kliniken Erlabrunn, Breitenbrunn, Germany

**Keywords:** active inference, candidate annotation architecture, computational psychiatry, early modern Iberia, human-centered AI, human-in-the-loop, intermediate representation layer, interpretable AI

## Abstract

Computational psychiatry has advanced formal accounts of individual prediction, affect regulation, and maladaptive rigidity, but it still has fewer clinically interpretable tools for representing social and institutional processes under collective stress. This Hypothesis and Theory article proposes a theory-driven candidate annotation architecture for this purpose. It defines five collective-process variables—defensive closure (DEF-C), collective anxiety load (ANX-C), integrative progression (PRO-C), punitive superegoic regulation (SUP-C), and moral frame rigidity (FRAME-C)—as theory-guided interpretive indicators for trained human annotation and later formal modeling. Drawing on Active Inference, PAD-S/CSA, Conceptual Metaphor Theory, Moral Politics, social defense theory, and threat-rigidity research, the framework specifies how historically or institutionally situated material can be parsed through explicit coding units, evidence spans, ordinal ratings, confidence markers, adjudication rules, variable-overlap handling, and prospective disconfirmation criteria. Early modern Iberia serves as a worked coding demonstration, with an internal contrast between layered plurality under constraint in al-Andalus and later confessional/inquisitorial consolidation; the Dutch Republic is added as a brief contrastive horizon. Because the framework is applied to historically mediated material, it treats anachronism, narrative uncertainty, interpretive plurality, and temporal delimitation as methodological constraints rather than residual problems. The article does not claim validated measurement status and does not report inter-coder reliability, fitted state-space estimation, or predictive performance. Its contribution is pre-validational and methodological: it provides an intermediate representation layer designed to make clinically interpretable collective-process hypotheses explicit, open to critical inspection, and suitable for future reliability testing, comparative coding, baseline comparison, and human-in-the-loop computational modeling.

## 1 Introduction: the representational gap

Computational psychiatry has advanced mechanistic accounts of learning, prediction, decision-making, and symptom formation, but clinically useful AI and computational modeling depend not only on model performance. They also depend on the intermediate variables through which complex human material is represented. The field now has many formal tools and increasingly powerful models, yet comparatively few clinically grounded variables that remain interpretable when analysis moves from the individual to the dyad, the group, the institution, and the wider social field (1–4).

PAD-S and CSA address this bottleneck at the level of psychotherapy by formalizing defense, anxiety/tolerance, progression, and self-attack/shame under explicit safety thresholds (5, 6). A related service-level heuristic shows the same principle in another domain: even an apparently straightforward descriptor such as “flat affect” becomes clinically useful only when it is treated as a context-sensitive capacity signal rather than a fixed trait label (7). The present paper asks whether the same representation-first discipline can be extended across scales to collective and institutional regulation without diagnosing societies as if they were patients.

The classical biopsychosocial and neurobiopsychosocial models remain indispensable, but they are substantially stronger at describing how social conditions affect the individual than at describing how social systems themselves may become carriers of affect regulation, defensive closure, moralization, and epistemic narrowing. Conversely, the social sciences and humanities often provide nuanced historical and cultural description, but not a clinically interpretable intermediate representation that can later be translated into annotation schemes, decision support, or computational models. This leaves a gap exactly where human-centered AI and clinically grounded innovation require the greatest clarity: at the interface between richly described human process and formal representation.

This article therefore addresses a deliberately bounded question: can a small set of clinically familiar process problems be reformulated as observable collective and institutional dynamics in a way that is transparent, annotatable, falsifiable, and ethically constrained? The paper proposes a candidate annotation architecture: a collective companion layer, explicit units of analysis, decision rules, ordinal coding options, and a staged validation roadmap. Early modern Iberia is used as a worked coding demonstration because it makes plurality, confessional consolidation, institutional surveillance, blood-purity classification, and exclusion unusually visible; the case logic distinguishes an internal Iberian contrast (al-Andalus versus later confessional/inquisitorial Spain) and pairs it with a brief Dutch Republic sketch as an external contrastive horizon. The case material illustrates coding logic and helps specify future validation requirements rather than serving as validation itself.

The article therefore occupies a deliberately intermediate position. It does not claim to have solved measurement, reliability, or model estimation; instead, it specifies the representational layer required before such work can be responsibly attempted. Its intended contribution is to make clinically interpretable collective-process variables explicit enough for later reliability testing, comparative coding, adjudication, baseline comparison, and formal modeling.

## 2 Scope, epistemic status, and non-goals

### 2.1 Epistemic status: annotation architecture rather than validated measurement framework

This article occupies a pre-validational epistemic position. DEF-C, ANX-C, PRO-C, SUP-C, and FRAME-C are defined as candidate annotation targets rather than as independently validated measurement constructs. The variables are theory-guided interpretive indicators intended for trained human annotation under explicit evidence, uncertainty, confidence, and adjudication rules; they should not be treated as directly observable natural kinds, emic categories of historical actors, or reliably separable variables before empirical testing. The contribution is therefore representational and methodological: it specifies a candidate annotation architecture that can make later reliability testing, discriminant-validity assessment, baseline comparison, and state-space or sequence modeling possible. Observed cues, coded representations, and downstream interpretation remain separate: source passages, events, and procedures supply evidence; DEF-C, ANX-C, PRO-C, SUP-C, and FRAME-C are human-coded representations of that evidence; and any clinical, institutional, or policy conclusion is a separate downstream judgment outside the present manuscript’s validation claims.

A further constraint concerns historically mediated evidence. The framework does not treat historical narratives as direct observations, but as interpreted, source-dependent reconstructions that already contain uncertainty. Applying clinically inspired process language to such material therefore introduces a second layer of uncertainty: the uncertainty of the historical record and the uncertainty of cross-domain interpretation. The proposed annotation architecture is intended to manage, not eliminate, this problem by requiring explicit evidence spans, confidence ratings, uncertainty codes, negative cases, and adjudication rules. In this sense, interpretive plurality is not bypassed; it is made visible, bounded, and open to later reliability testing.

### 2.2 Cross-level analogy, translation rule, and non-goals

The most important methodological distinction in this paper is between literal identity, unconstrained analogy, and possible cross-level structural correspondence as a future validation target. The article does not claim that societies have brains, that states have unconscious fantasies, or that institutions can be clinically diagnosed. It proposes a disciplined cross-level process analogy. Stronger structural-correspondence claims would require evidence that the relations among variables remain stable across individual, dyadic, group, institutional, and historical levels of analysis; this manuscript treats such correspondence as a difficult empirical target, not as an established premise.

**Figure 1:**
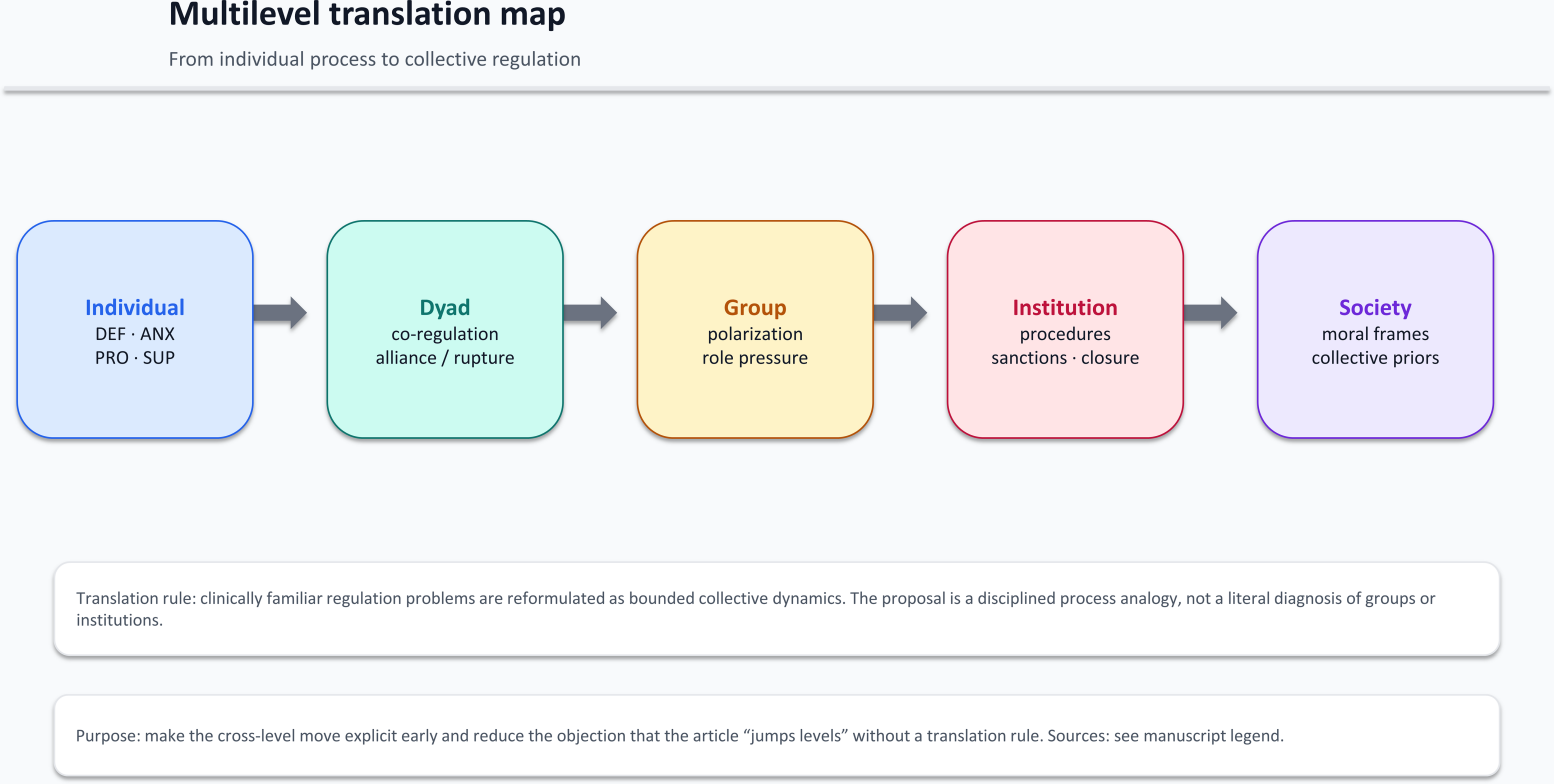
Multilevel translation map. Schematic transition from individual process to dyadic, group, institutional, and societal regulation. The image clarifies that the manuscript proposes a disciplined cross-level analogy rather than literal identity between levels.

The basic translation rule is narrower. Persons regulate uncertainty through embodied perception, affect, defense, and action. Groups and institutions regulate uncertainty through shared narratives, norms, sanctions, roles, rituals, documents, procedures, and symbolic boundaries. The substrates differ, but some regulation problems may recur in structurally comparable ways: closure versus revisability, threat load versus tolerance, punitive moralization versus integrative learning, and frame rigidity versus frame flexibility. **Figure 1** visualizes this translation rule and makes the cross-level move explicit without treating groups or institutions as clinical patients.

The framework is thus a candidate annotation architecture and intermediate representation layer, not a policy recommendation or an autonomous classification system. It is designed to support disciplined human-in-the-loop annotation, comparative case coding, reflective supervision, education, and later formal modeling only after reliability and measurement validity have been tested. It is not designed to generate automated scores of “dangerous cultures,” to pathologize nations, to replace historical explanation, or to justify punitive prediction regimes. Its proper use is interpretive, comparative, reflective, and human-final. **Box 1** summarizes these non-goals and safeguards before the theoretical anchors are introduced.

Box 1 **Non-goals and safeguards**

• The framework does not diagnose nations, religions, cultures, or historical populations as clinical patients.

• Early modern Iberia is used as a worked coding demonstration, not as proof of the model and not as a moral ranking of civilizations.

• The paper proposes a disciplined process analogy and possible cross-level structural-correspondence conditions; it does not claim identical ontologies across levels.

• Codes attach to bounded evidence spans or episodes, not to whole peoples, civilizations, or religious traditions.

• The framework is not an autonomous decision system and should not be used for collective risk scoring.

• Human interpretation, adjudication, and contestability remain final.

## 3 Theoretical anchors

### 3.1 Active inference and socially shared generative models

The Active Inference tradition offers a useful formal scaffold for the present argument. In the free-energy and predictive-processing family of models, the brain is understood less as a passive receiver of stimuli than as an inference engine that predicts, samples, updates, and acts to reduce uncertainty (1–3). This shift matters because it replaces a largely reactive picture of human functioning with one organized around priors, precision, action, and prospective regulation.

Cheadle and colleagues make the social extension explicit by arguing that brains are enculturated in situations and, through such dynamics, share models of the world with one another, enabling collective realities (4). This provides a disciplined way to think about institutions: not as minds, but as durable, socially distributed arrangements that stabilize what counts as plausible, threatening, sacred, deviant, or revisable. In this sense, collective regulation can be understood as uncertainty management through culturally reinforced and institutionally enacted priors.

### 3.2 PAD-S, CSA, and safety-gated process representation

PAD-S and CSA were developed to make psychotherapy micro-decisions clinically interpretable and machine-readable without flattening them into generic sentiment or dialogue acts (5, 6). Both frameworks formalize the clinician’s real-time tracking of defense, anxiety/tolerance, progression, and self-attack/shame, while explicit thresholds constrain admissible intervention dose. The later PAD-S/CSA shared representation paper further isolated the representational role from school-specific policies and clarified the observation–representation–policy distinction (8).

This matters for the present paper for two reasons. First, it shows that clinically meaningful process variables can be formalized without becoming reductionistic. Second, it provides a methodological precedent for defining clinically interpretable coordinates before fitting predictive models. The present article extends this logic across levels by proposing a collective companion layer. It does not extend therapy technique to societies; it extends the representation-first discipline of clinically meaningful state variables to collective material.

### 3.3 Moral metaphor, moral politics, and FRAME-C

Conceptual Metaphor Theory provides the bridge from individual cognition to collective moral meaning. Lakoff and Johnson argued that metaphors are not merely ornamental language but structure abstract domains through more concrete source domains (9, 10). In politics, the nation-as-family metaphor is especially relevant. Lakoff’s strict-father and nurturant-parent models were initially criticized as essayistic, but later empirical work by Feinberg, Wehling, and colleagues demonstrated that family-based moral models predict political stances and that biconceptual individuals can shift under moral framing (11, 12).

For the current model, moral metaphor is not decoration; it is an observable vehicle of collective priors. A strict-order or purity-oriented frame can make discipline, obedience, boundary enforcement, and punishment feel morally intuitive. A care-centered or plurality-preserving frame can make protection, repair, empowerment, and revisability feel morally intuitive. FRAME-C therefore needs to be operationalized as both direction and rigidity: a frame may be strict but flexible, care-centered but weak, or mixed and unstable. Direction alone is not coded as pathology or virtue; the clinically relevant question is whether the frame becomes rigid, unrevisable, punitive, or evidence-resistant under threat. This distinction is relevant for later coding and modeling because moral direction and rigidity need not covary.

### 3.4 Social character and institutional defense

Fromm’s analytic social psychology supplies the social bridge that is otherwise missing in strictly clinical theories. In his posthumous English collection Beyond Freud, Fromm develops the move from individual to social psychoanalysis and treats the individual as socially situated rather than psychologically isolated (17). This prevents the false choice between “society determines everything” and “everything is intrapsychic.” Fromm’s Escape from Freedom is also relevant because it formulates the authoritarian temptation as a flight from uncertainty, isolation, and freedom itself (18).

Menzies’s classic study of a hospital nursing service showed that organizations can function as social defenses against anxiety (19). Mentzos extended this line toward interpersonal and institutionalized defense (20), and Bion’s work on group basic assumptions clarifies how groups may organize dependency, fight-flight, or other regressive patterns under stress (21). Together, these traditions justify speaking of institutions as anxiety-regulating systems while also requiring caution: institutional defense is not a diagnosis of an institution; it is a hypothesis about the function of observable procedures, roles, and symbolic practices.

### 3.5 Structural fragility, empathy, and regression

The psychodynamic literature helps specify what changes under stress. Frederickson’s contribution on empathy is especially useful because it defines empathy not as warmth but as calibration to what another person can bear (22). What is experienced as supportive in one patient can be experienced as invasive in another. This dose-matching logic maps naturally onto collective settings: what a social system can tolerate under low threat may become intolerable under high threat, and attempts at pluralism may be experienced as fragmentation rather than growth.

A related clinical-political formulation develops this point through the concept of symbolization thresholds: under affective load, the capacity to transform raw pressure into shareable meaning may narrow, giving way to concrete action, rigid certainty, dissociation, or persecutory simplification (23). In the present article, this idea is not used to diagnose political communities, but to clarify why ANX-C and FRAME-C rigidity matter as coding targets: they mark conditions under which shared meaning may become less revisable and more vulnerable to defensive closure. Conversely, the repair side of the same logic—co-symbolizing dialogue—helps specify the functional meaning of PRO-C as plurality-preserving revision rather than merely the absence of coercion.

Ogden’s reformulation of depressive, paranoid-schizoid, and autistic-contiguous modes further supports a process rather than stage model (24). These modes of organizing experience coexist and may dominate under different conditions. The depressive mode allows ambivalence, history, guilt, reparation, and subjectivity. The paranoid-schizoid mode privileges splitting, projection, and persecutory simplification. Kohut and Kernberg add bounded concepts of selfobject regulation, idealization, primitive defense, and grandiose stabilization (25, 26). Applied cautiously, these concepts help formulate hypotheses about why leaders, institutions, or nation-symbols may become repositories of collective idealization in times of threat; they are not used here to diagnose societies or historical populations.

### 3.6 Adjacent literatures on threat and institutional response

The framework is not intended to replace organizational or political-science accounts of threat. It sits adjacent to threat-rigidity theory in organizational behavior, which describes restriction of information processing and constriction of control under threat across individual, group, and organizational levels (27). It also overlaps with organizational threat-response research, which examines how organizations interpret, categorize, and respond to threats under uncertainty (28). Political-psychological work on motivated social cognition similarly links threat, system instability, intolerance of ambiguity, need for closure, and reduced integrative complexity to more rigid political attitudes (29, 30).

**Figure 2:**
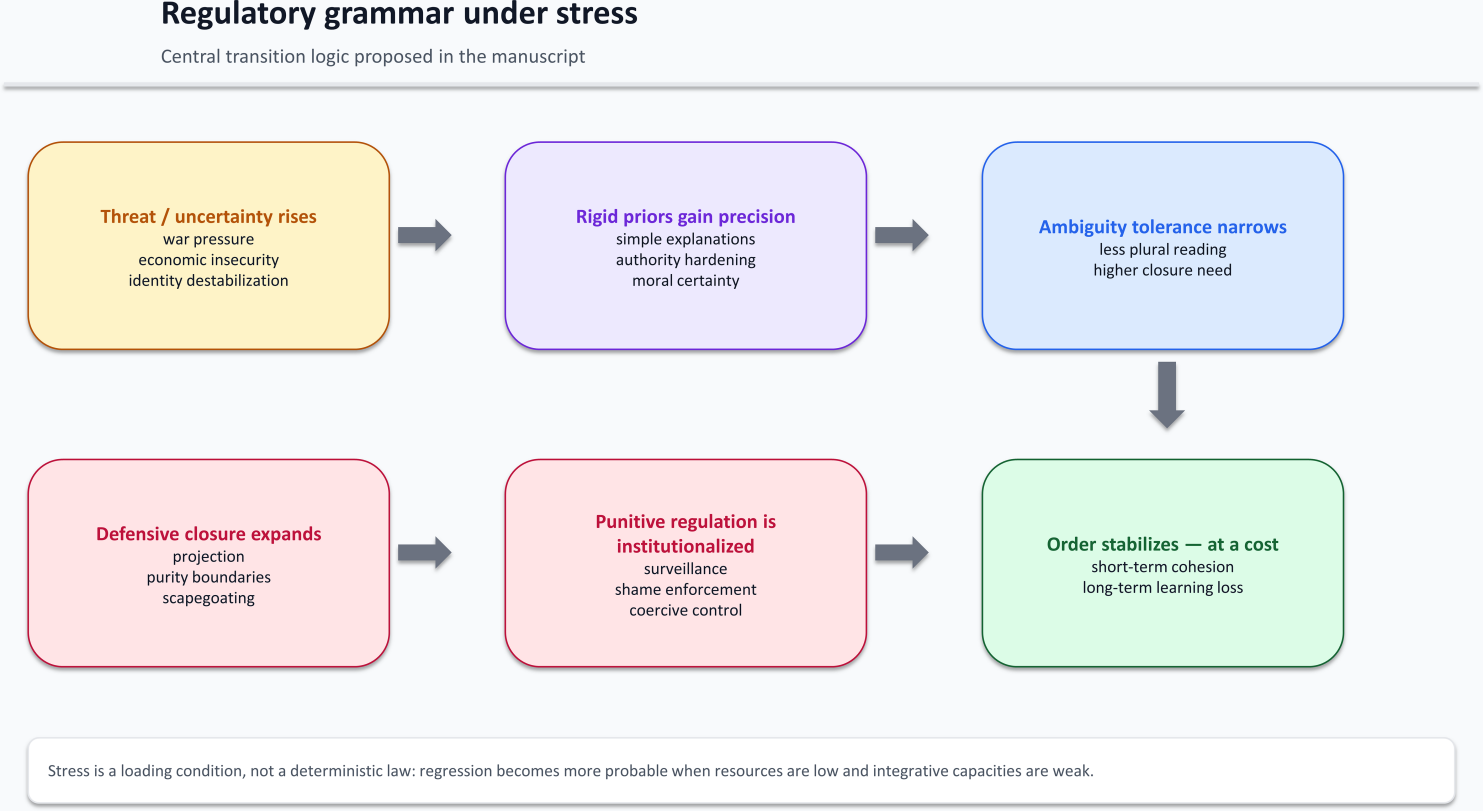
Regulatory grammar under stress. Threat load increases the probability of frame rigidity, narrowed ambiguity tolerance, defensive closure, and punitive regulation. The transition is probabilistic, not deterministic, and buffering conditions may preserve PRO-C under stress.

The contribution of the present proposal is therefore translational rather than discovery-based: it specifies these phenomena as clinically interpretable, process-oriented companion variables that could be coded across psychotherapy, organizational, institutional, and historical material, while keeping observation, representation, and policy separate. **Figure 2** condenses the proposed threat-to-closure grammar and explicitly presents it as probabilistic rather than deterministic.

## 4 Positioning: a candidate intermediate representation layer between theory and model

The present proposal should be located between, rather than above, several adjacent modeling traditions. Active Inference and related social extensions offer a formal language for priors, precision, coordination, and transition dynamics (1–4), while PAD-S/CSA provide a clinically grounded process grammar for safety-gated psychotherapeutic decision-making (5, 6, 8). Observer-based psychotherapy process coding shows how relational microprocesses can be rated without reducing them to symptom counts (31). MIP/MIPVU and critical metaphor analysis provide procedures for identifying metaphorical framing in texts (14–16). V-Dem-style expert-coded measurement and event-coding projects demonstrate how complex institutional or political material can be translated into ordinal or event-level data under explicit uncertainty and coder procedures (32, 33).

The collective companion layer differs from these traditions by combining their respective strengths at a narrower intermediate level. It does not replace Active Inference with a new formal theory, does not replace historical interpretation with automated coding, and does not replace institutional sociology or political science with psychodynamic metaphor. Its contribution is translational: DEF-C, ANX-C, PRO-C, SUP-C, and FRAME-C are proposed as theory-guided interpretive indicators that preserve psychodynamic and social-theoretical meaning while becoming explicit enough for annotation, adjudication, uncertainty handling, and later model comparison. In this sense the article adds a candidate bridge layer, not a validated measurement system or universal explanatory theory.

The framework is particularly suitable for material in which threat, moral framing, boundary work, sanction, and institutional revision leave traceable textual or event-based evidence: psychotherapy transcripts, organizational communication, clinical-service documents, political or religious texts, reform debates, procedural records, and historical event sequences. It is less suitable for sparse sources, one-sided moral judgment, automated group risk scoring, or settings where the investigator cannot distinguish observed cues from totalizing cultural explanation. Its appropriate use is comparative, reflective, and human-final: it can support clinical supervision, education, institutional reflection, and human-centered computational modeling of regulation under stress rather than retrospective blame, cultural diagnosis, or automated prioritization of groups.

**Figure 3:**
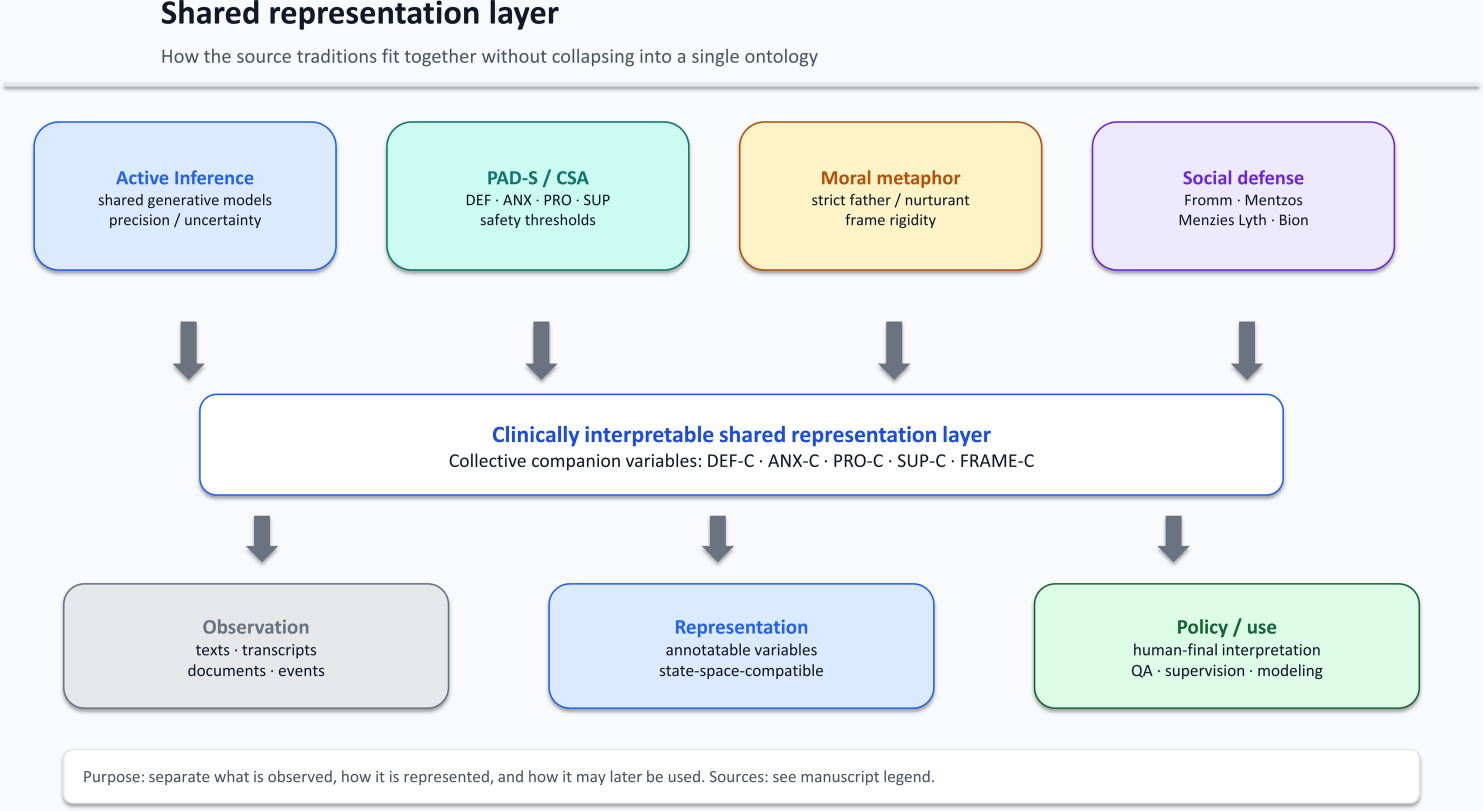
Candidate intermediate representation layer. Active Inference, PAD-S/CSA, moral metaphor, social defense theory, and adjacent threat-response literatures are mapped into an interface that separates observation, representation, and policy without collapsing them into a single ontology.

Taken together, these literatures support a theory-guided, clinically interpretable intermediate representation layer between formal computational psychiatry, social Active Inference, and metaphor- or framing-oriented approaches to collective meaning. Generative and active-inference-based accounts are strongest when latent inference, learning, precision, and transition processes are formalized; metaphor and framing approaches show how abstract social order is stabilized through recurrent semantic patterns; and observer-based process coding demonstrates how clinically meaningful intermediate variables can be defined before fully predictive models are fitted (1–4, 9–16, 31). The specific contribution of the present article is therefore not to merge these traditions into a total theory, but to translate their overlap into human-readable, annotatable process variables for collective threat regulation, moral framing, punitive closure, and integrative revision. **Figure 3** depicts this candidate shared representation layer and makes the observation–representation–policy separation visually explicit. This positioning motivates the candidate annotation architecture introduced below.

## 5 Candidate annotation architecture and collective companion variables

### 5.1 Translation rule and variables

Operationally, this positioning is translated into a collective companion layer consisting of five variables. DEF-C denotes collective defensive closure, externalization, projection, or boundary policing. ANX-C denotes collective threat load and ambiguity intolerance. PRO-C denotes integrative learning and plurality-preserving adaptation. SUP-C denotes punitive moralization, shame enforcement, or coercive obedience testing. FRAME-C denotes the dominant moral frame and its rigidity. The variables are companion variables to PAD-S/CSA, not identical copies of individual clinical states. **Table 1** gives a compact overview of the variables, core coding cues, and key cautions; the extended theoretical crosswalk is provided in **Supplementary Table 1**.

**Table 1:** Candidate collective-process variables at a glance.

Variable	What to code	Key caution
DEF-C	Externalization, projection, scapegoating, contamination semantics, exclusion of ambiguous or liminal actors, and ritualized boundary policing.	Code only when disturbance is attributed to an externalized object in a way that reduces complexity; ordinary boundary maintenance is insufficient.
ANX-C	Explicit or contextually grounded threat load, ambiguity intolerance, emergency language, breakdown, moral panic, or repeated uncertainty markers.	Do not back-code anxiety from repression or punishment alone; distinguish no evidence (0) from insufficient information (NA/unclear).
PRO-C	Revision, repair, reintegration, negotiated coexistence, procedural adaptation, and plurality-preserving conflict processing.	Code only when integration or revision is a legitimate solution; tactical exception or uncontrolled fragmentation is insufficient.
SUP-C	Punitive moralization, shame enforcement, public obedience testing, denunciation, exemplary sanction, or coercive correction.	Technical sanction without moral loading is insufficient; distinguish punitive regulation from externalizing DEF-C.
FRAME-C	Dominant moral frame and rigidity: strict-order/purity, authority, unity, care, repair, protection, plurality, and whether the frame is revisable.	Code direction and rigidity separately; isolated moral phrases are insufficient unless they become structure-bearing.

Operationally, the primary coding unit is not a society, religion, or nation. The primary coding unit is a bounded text segment, institutional episode, or event sequence. Coders do not infer collective motives. They code observable markers of framing, threat, externalization, sanction, and adaptive revision. This distinction is the main safeguard against circular psychohistorical interpretation.

### 5.2 Annotation architecture

**Figure 4:**
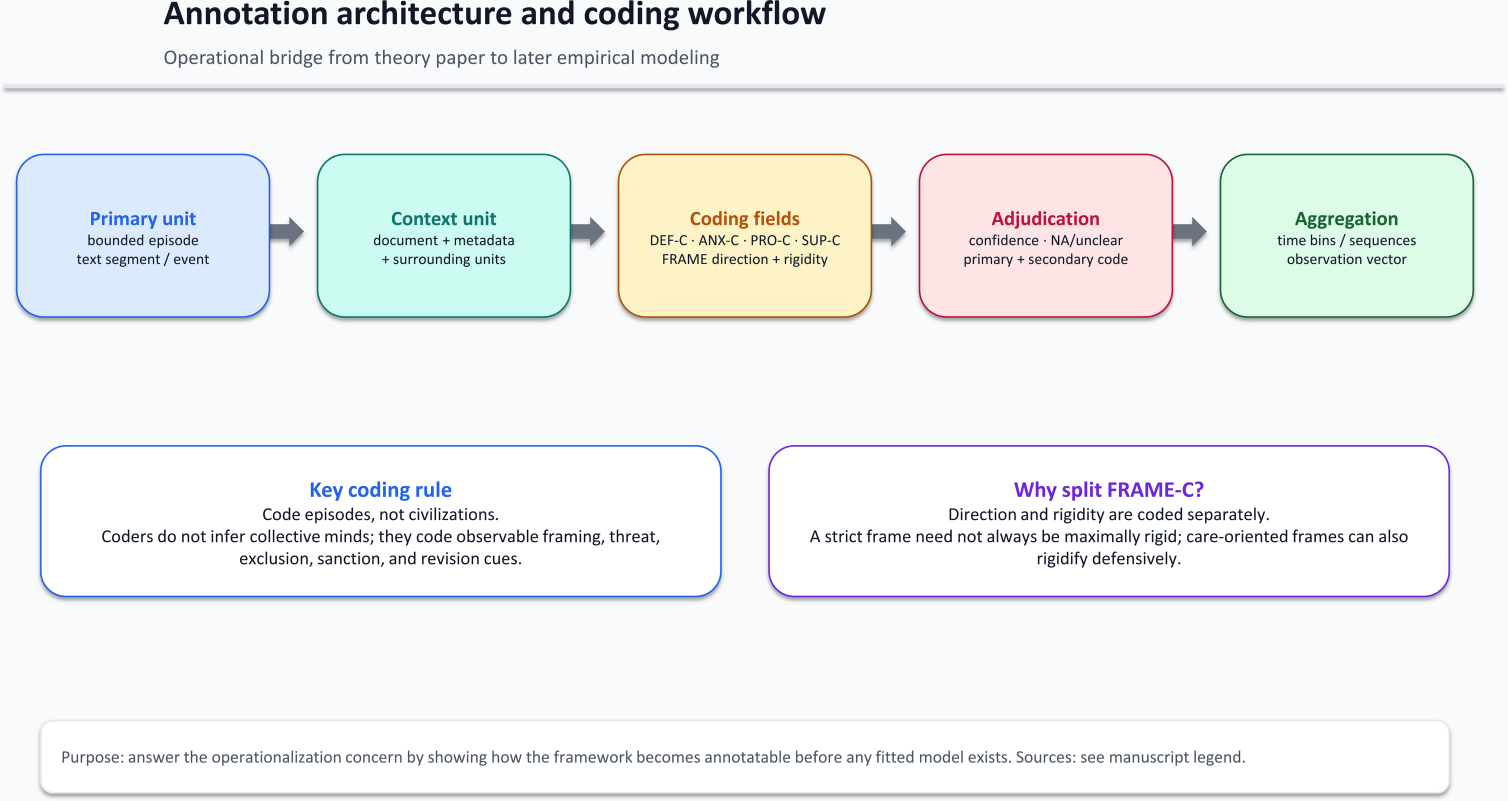
Annotation architecture and coding workflow. Episode-based coding structure showing primary unit, context unit, coding fields, adjudication, aggregation, and the separation between observable cues, interpretive indicators, and later modeling.

The proposed operationalization should be read as a specimen annotation architecture and as a prerequisite for a later coding manual. The variables remain theory-guided interpretive indicators, and this article does not yet demonstrate that independent coders can apply the distinctions reliably. The purpose of the operational layer is to make the interpretive variables sufficiently explicit for training, evidence-span annotation, NA/unclear handling, evidence-clarity confidence ratings, primary/secondary coding, adjudication, and later reliability testing. These procedures do not remove interpretive variance, but they prevent it from remaining tacit: disagreement, uncertainty, and boundary cases become part of the object of documentation rather than failures of the method. **Figure 4** summarizes this coding workflow, while **Supplementary Tables S2–S5** provide the extended annotation architecture, specimen codebook, ordinal encoding scheme, and state-space-compatible encoding options for future empirical piloting.

### 5.3 Extended codebook and ordinal encoding

The detailed specimen codebook is provided in **Supplementary Table 3**, and the ordinal encoding scheme and confidence field are provided in **Supplementary Table 4**. The examples are synthetic and paraphrased; they clarify coding boundaries and prepare a later pilot study rather than introducing new historical evidence. This redistribution keeps the main text readable for clinical, cultural, and social-scientific readers while preserving the operational detail needed for future reliability testing.

### 5.4 Decision hierarchy and adjudication logic

The codebook should be applied using a simple decision hierarchy. First, coders assess ANX-C from the unit and its context, without back-coding anxiety from the presence of repression or punishment. Second, they identify whether a structure-bearing moral frame is present and, if so, code FRAME-C direction and rigidity separately. Third, they code the dominant immediate regulatory function: DEF-C when externalization and boundary maintenance dominate; SUP-C when shame, punishment, and obedience testing dominate; PRO-C when revision, inclusion, and plurality-preserving adaptation dominate. Secondary codes are assigned only when separate evidence spans support an additional function. A primary code should be assigned whenever the unit is used for transition or sequence modeling.

Disagreement should be treated as data, not noise to be hidden. Pre-adjudication and post-adjudication labels should both be archived. Adjudication is mandatory for differences greater than one ordinal level, sign changes in FRAME-C direction, and DEF-C/SUP-C or PRO-C/SUP-C conflicts. The immediate institutional function should guide resolution: boundary-maintenance favors DEF-C; moral punishment and shame favor SUP-C; rule revision or reintegration favors PRO-C.

### 5.5 Variable overlap and discriminant validity

Partial overlap between DEF-C, SUP-C, and FRAME-C is expected rather than denied. Boundary-policing, punitive moralization, and rigid moral framing may co-occur in the same historical, institutional, or organizational episode. The framework therefore does not assume that the variables are naturally independent. It treats discriminant validity as a future validation target. Primary and secondary coding, evidence spans, confidence ratings, NA/unclear categories, and adjudication rules are introduced precisely to make overlap visible and testable rather than to hide it.

### 5.6 State-space-compatible encoding options

State-space-compatible encoding options are presented here as a future modeling route, not as evidence of a fitted computational model. They follow from the proposed ordinal encoding and remain conditional on later reliability and measurement-validity testing. The main text therefore keeps the notation deliberately light. Let y_t = [DEF-C_t, ANX-C_t, PRO-C_t, SUP-C_t, FRAMEdir_t, FRAMErig_t] denote the observed ordinal coding vector at time t; let z_t denote a latent collective regulation state estimated from observed units; and let u_t denote contextual inputs such as war pressure, scarcity, reform episodes, institutional crises, or external threat. Extended encoding strategies are provided in **Supplementary Table 5**.

Observation vector:

y_t = [DEF-C_t, ANX-C_t, PRO-C_t, SUP-C_t, FRAMEdir_t, FRAMErig_t].

Ordinal measurement sketch:

P(y_itj ≤ c | z_tj) = logit^{-1}(τ_jc − λ_j z_tj).

Transition sketch: z_t = A z_{t−1} + B u_t + ε_t.

The main later-testable expectation is not that this article already estimates A or B, nor that the proposed variables have already achieved measurement validity. It is that, if the annotation architecture demonstrates adequate reliability, increases in ANX-C and FRAME-C rigidity should make higher DEF-C and SUP-C, and lower or more constrained PRO-C, more probable unless buffering conditions are present. This is compatible with human-in-the-loop annotation first, probabilistic aggregation second, and only then latent, sequence, multi-agent, or state-space modeling. **Supplementary Table 5** lists encoding strategies and their corresponding strengths and limits.

### 5.7 Prospective disconfirmation criteria

A theory paper should state what would count against the proposal before empirical testing begins. **Table 2** specifies four prospective disconfirmation criteria that would weaken or force revision of the architecture.

**Table 2:** Prospective disconfirmation criteria.

Claim at risk	What would count against it	Minimum evidence needed
Variables are usable analytic targets.	Coders cannot distinguish DEF-C, SUP-C, PRO-C, ANX-C, and FRAME-C after training and manual revision, especially DEF-C vs. SUP-C or PRO-C vs. absence of punishment.	Double-coded pilot units, adjudication logs, reliability estimates.
Threat plus rigid framing predicts closure.	Contrastive corpora repeatedly show high ANX-C and high FRAME-C rigidity without increased DEF-C/SUP-C or constrained PRO-C, unless buffering conditions are specified.	Sequenced document/event coding and contextual threat markers.
The representation adds value beyond simple labels.	Ideology-only, event-count-only, sentiment-only, or punitive-only baselines perform as well as or better than the five-variable process representation.	Model comparison using the same corpus and comparison design.
The model is not a moral ranking device.	Cases collapse into the same evaluative story regardless of observed cues, suggesting unfalsifiable moral interpretation.	Contrastive cases, negative examples, and transparent adjudication records.

## 6 Worked coding demonstration: early modern Iberia

### 6.1 Why Iberia: visibility of coding logic, not proof of theory

Early modern Iberia is useful as a worked coding demonstration because it makes plurality, confessional consolidation, institutional surveillance, blood-purity classification, and exclusion unusually visible within a single broad historical field. The temporal scope of the demonstration is deliberately limited. It does not treat Iberian history as a whole as an object of psychological explanation. Instead, it focuses on broad early modern transition dynamics surrounding the consolidation of confessional and inquisitorial governance after the late fifteenth century. Al-Andalus functions as a historically prior internal contrast, and the Dutch Republic as a brief external contrastive horizon. This temporal delimitation is not intended to define a closed periodization of Iberian history; it limits the demonstration to a tractable interval in which the proposed variables become especially visible.

**Figure 5:**
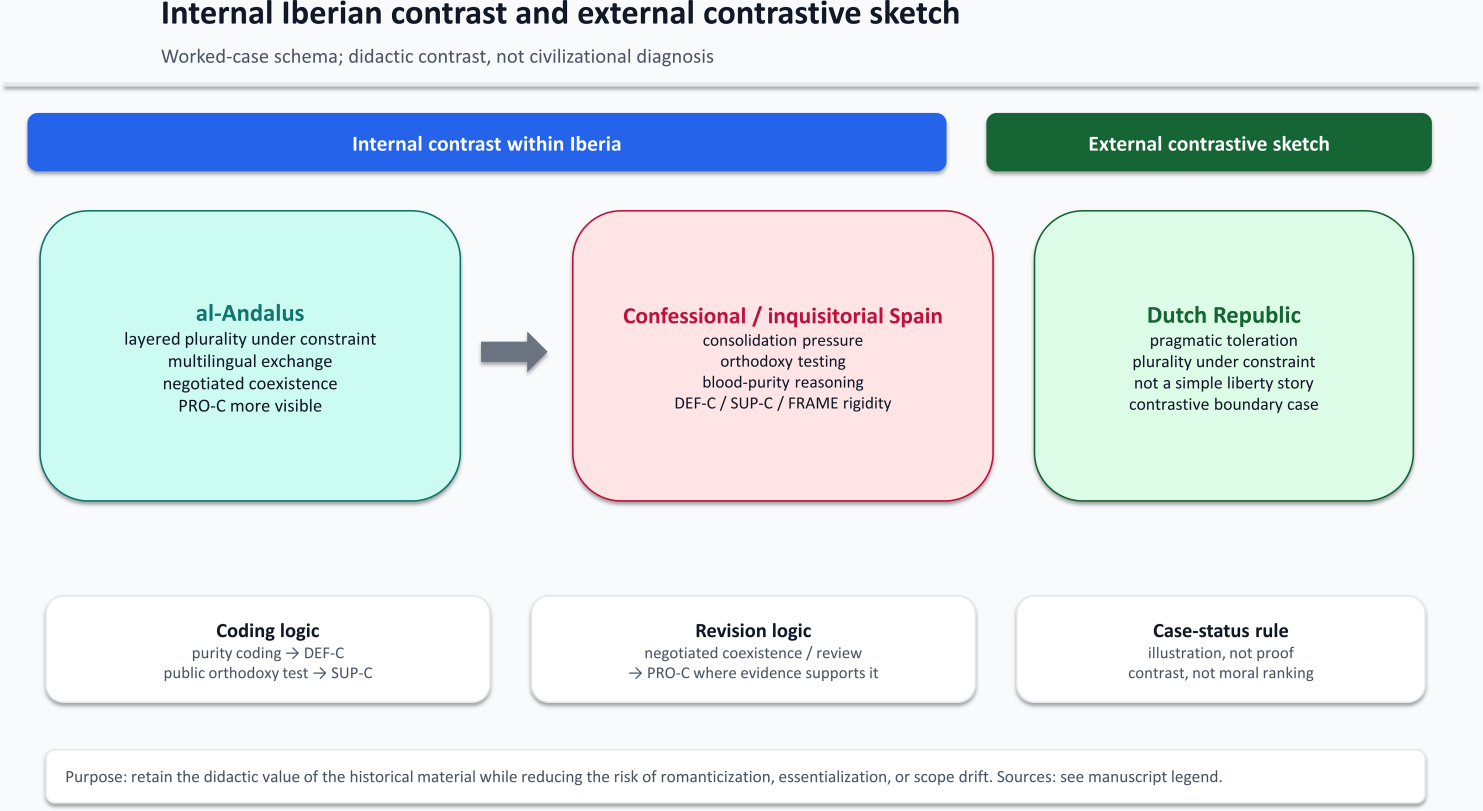
Internal Iberian contrast and external contrastive sketch. Al-Andalus is shown as layered plurality under constraint; later confessional/inquisitorial Spain as consolidation pressure, punitive closure, and genealogized exclusion; the Dutch Republic is added as a brief external sketch of pragmatic toleration and negotiated coexistence. The image is didactic, not civilizationally diagnostic.

The case is staged as an internal contrast between layered plurality under constraint in al-Andalus and later confessional/inquisitorial consolidation. This contrast is heuristic rather than civilizational. The point is not to contrast a “good” Islamic past with a “bad” Christian present, nor to diagnose Spain. Historiography on convivencia has long moved beyond romantic idealization; coexistence in al-Andalus and Christian Iberia was real but uneven, conflictual, and historically contingent (36, 37). Because source genres, archival density, translation, and historiographic framing differ across settings, the examples below are treated as conditional coding demonstrations tied to observable cues, not as claims about deeper historical truth. **Figure 5** summarizes the internal Iberian contrast and the external Dutch Republic horizon as a didactic coding schema, not as a moral ranking.

The case therefore functions as a transparent demonstration of coding logic rather than as a validation study. A full test would require independent coding, reliability estimates, discriminant-validity assessment, baseline comparison, and at least one external contrastive case. Here Iberia is used to show how the proposed architecture would parse historically familiar material: what would count as a coding cue, what would remain uncertain, and where a later validation study would need to put the model at risk.

### 6.2 Historiographic grounding and internal Iberian contrast

Seen through this contrast logic, al-Andalus and other mixed Iberian settings can be treated as a comparatively more plural configuration, albeit one marked by hierarchy, conflict, and constraint rather than by egalitarian harmony. Akasoy and Soifer are useful precisely because they resist the myth of frictionless convivencia while still allowing historians to describe uneven but real forms of multilingual, interfaith, and institutionally mediated coexistence (36, 37). In the present framework, such material does not automatically count as high PRO-C; it counts only where plurality is procedurally managed, negotiated, or left revisable.

Later confessional/inquisitorial Spain marks a different regulatory configuration. Hossain’s review of inquisitorial studies and Kamen’s synthetic account show why the Inquisition should be understood institutionally rather than as a merely symbolic synonym for cruelty (38, 39). García-Arenal and Wiegers on the Morisco expulsion, together with García-Arenal’s recent discussion of blood, race, and religion, clarify how genealogized suspicion and blood-purity reasoning could narrow revisability and convert ambiguous membership into inherited threat (40, 41). The internal Iberian contrast is therefore not civilization versus civilization. It is a contrast between more layered plurality under constraint and a later regime of confessional consolidation, punitive closure, and genealogized exclusion.

### 6.3 Micro-coding examples

The following examples are synthetic, paraphrased specimen examples. They are included to illustrate how a coder could differentiate variables, not to claim direct quotation from a particular archival document. A compact boundary example is useful: blood-purity reasoning that treats ambiguous membership as inherited and unrevisable would primarily support high FRAME-C rigidity and DEF-C, whereas a technical doctrinal clarification without shame, sanction, or externalized causality would not automatically support SUP-C or DEF-C. Additional micro-coding examples for al-Andalus, confessional/inquisitorial Spain, and the Dutch Republic are provided in **Supplementary Table 6**.

### 6.4 What the internal contrast illustrates

Read contrastively, the Iberian material juxtaposes a comparatively more plural and partly revisable configuration—still unequal, unstable, and historically bounded in al-Andalus and other mixed settings—with a later configuration in which confessional and dynastic consolidation increasingly narrowed plurality. From the present framework, the shift is less intelligible as a clash of timeless essences than as a reorganization of collective regulation under stress. Once confessional unity became a core political task, plurality itself could be reframed as threat. In Lakoffian terms, the political field moved toward a stricter family logic in which one crown, one faith, and one law were increasingly coded as moral necessities rather than administrative options (9–12).

The Inquisition can then be interpreted as an institution of punitive regulation rather than merely as an instance of cruelty. In the proposed codebook, this corresponds to elevated SUP-C when moral judgment is linked to public shame, sanction, and obedience testing. It corresponds to DEF-C when ambiguity is located in liminal persons or groups and converted into an externalized threat. The blood-purity logic further increases FRAME-C rigidity because difference is no longer treated as a temporary belief or practice but as a genealogically transmissible sign. PRO-C becomes harder because integration is no longer straightforwardly imaginable. This internal Iberian contrast sharpens coding expectations, but it does not remove the need for an external contrastive horizon.

### 6.5 External contrastive horizon: Dutch Republic

A brief external contrastive horizon is the Dutch Republic. Kaplan’s account of religious conflict and the practice of toleration in early modern Europe is especially useful because it treats toleration as practical, fragile, and often local rather than as modern liberal harmony (42). That makes the Dutch material suitable as a short sketch rather than a new fully developed case. The present framework would lead us to expect that where institutional arrangements preserve negotiated coexistence, compartmentalized plurality, and revisable local compromise, PRO-C should remain more visible and SUP-C less totalizing than in cases where difference is genealogized as inherited taint.

Such a comparison creates disconfirmation risk in a way that a single worked case cannot. If high threat, flexible or only moderately rigid framing, and sustained PRO-C can coexist without rising DEF-C/SUP-C, the proposal must specify buffering conditions rather than simply repeating that threat causes rigidity. If, by contrast, high threat plus rigid frame direction and rigidity repeatedly produce closure and punitive enforcement across cases, the framework gains credibility. The point is not to force al-Andalus, confessional/inquisitorial Spain, and the Dutch Republic into a moral binary, but to establish explicit contrastive coding conditions.

### 6.6 What the demonstration enables and what remains untested

The demonstration’s role is bounded but useful. It shows how text-rich and institution-rich historical material could be parsed by the proposed annotation architecture under explicit evidence, uncertainty, and adjudication rules. It also clarifies which claims remain for future work: inter-coder reliability, discriminant validity, predictive validity, causal explanation, and state-space estimation. This boundary is part of the manuscript’s design. The present article offers a disciplined route from theory to annotatable representation; subsequent studies must test whether the variables are reliably separable, comparatively useful, and computationally modelable.

## 7 Relevance for human-centered computational psychiatry and AI in mental health care

The present proposal is intended as a representational contribution to human-centered computational psychiatry. Its main contribution is not a new historical interpretation of Iberia, but a candidate way to make clinically meaningful collective-process variables explicit enough for annotation, comparison, and later modeling. This matters for human-centered AI because the main problem in many current systems is not merely accuracy; it is that the system may learn categories that clinicians, historians, and service leaders would not trust as the basis for high-stakes interpretation. A clinically interpretable companion layer provides a more responsible target than generic sentiment, ideology labels, or decontextualized risk categories.

Several use cases follow, each conditional on later validation. First, the framework can support human-in-the-loop annotation of psychotherapy transcripts, organizational documents, institutional communications, and political texts. Second, it can support training and reflective supervision by making regressive group processes more discussable in process terms rather than in purely moral or polemical ones. Third, it opens the possibility of multi-agent modeling in which threat load, frame rigidity, and punitive feedback are simulated and compared under different assumptions about tolerance and frame flexibility.

The proposal is aligned with current ethics and governance concerns in AI for health. WHO guidance emphasizes ethics, human rights, transparency, accountability, safety, and human oversight in the design, deployment, and use of health AI (43, 44). A framework like the present one should therefore never be used to generate automated scores of cultures or groups. Its function is interpretive, reflective, and preventive. It is most useful when it slows overconfident labeling, preserves contestability, and makes it easier for human experts to compare explanations rather than harder.

## 8 Validation roadmap

**Figure 6:**
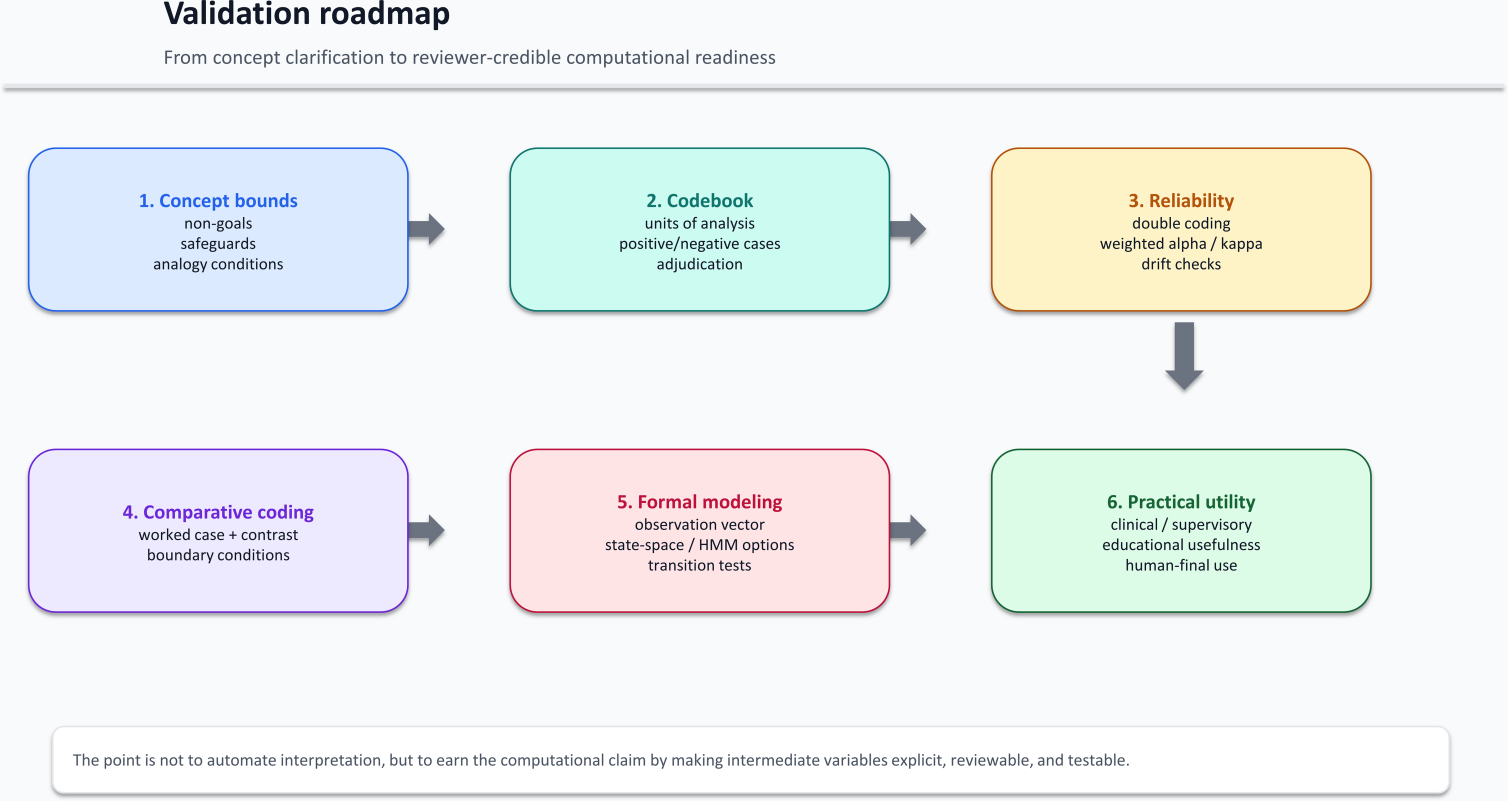
Validation roadmap. Conceptual clarification, codebook development, reliability testing, comparative coding, formal modeling, and practical utility are shown as staged validation targets rather than completed empirical results.

A candidate annotation architecture of this kind should be evaluated in stages rather than accepted or rejected as a global theory. The roadmap clarifies what the present pre-validational contribution enables: a move from conceptual specification to pilot annotation, reliability testing, comparative coding, baseline comparison, formal modeling, and practical utility assessment. **Figure 6** visualizes this staged roadmap, **Table 3** gives a compact main-text version, and **Supplementary Table 7** provides the extended validation protocol without implying that validation has already been achieved.

**Table 3:** Minimal validation roadmap.

Stage	Purpose	Minimum output
1. Codebook readiness	Test whether variables are intelligible, bounded, and usable by clinical, historical, and computational readers.	Revised manual with examples, negative cases, NA rules, confidence ratings, and adjudication logic.
2. Pilot reliability and discriminant validity	Test whether independent coders can apply the variables and whether DEF-C, SUP-C, PRO-C, ANX-C, and FRAME-C remain separable.	Double-coded pilot, weighted alpha/kappa, disagreement typology, and codebook revision log.
3. Comparative coding	Test whether the architecture travels across internally and externally contrastive cases without forcing moral binaries.	Iberia plus at least one external contrastive corpus with documented sampling and baseline labels.
4. Formal modeling readiness	Test whether coded sequences support interpretable aggregation, sequence modeling, or state-space modeling better than simpler baselines.	Baseline comparison, model specification, uncertainty reporting, and failure cases.
5. Practical and ethical utility	Test whether the framework improves human-final reflection without stigmatizing groups or automating political judgment.	Use-case review, safeguards, non-use criteria, and human oversight procedure.

Reliability thresholds should be pre-specified. A conservative plan would treat α or κ ≥ .80 as acceptable for substantive use, .67–.79 as tentative and requiring adjudication or revision, and < .67 as evidence that the variable or codebook requires redesign. These thresholds should not be treated as mechanical cutoffs; they are practical safeguards for a framework that aims to be clinically meaningful and computationally tractable.

## 9 Discussion

### 9.1 What the framework contributes

The proposed framework offers three main gains. First, it extends the neurobiopsychosocial model beyond the isolated individual without giving up clinical interpretability. Second, it translates psychodynamic process concepts into variables that can be related to Active Inference, moral framing, institutional defense, and organizational threat-response research. Third, it shows how historical material can be used as a worked coding demonstration with both internal and external contrast conditions rather than as a stage for moral condemnation or speculative diagnosis.

The operationalization is central to this contribution, but its status remains explicitly provisional. DEF-C, ANX-C, PRO-C, SUP-C, and FRAME-C are not presented as already validated natural kinds or independently established measurement constructs. They are candidate annotation targets. Their value depends on whether they can be coded reliably, distinguished from simpler labels, separated sufficiently in discriminant-validity work, used across contrastive cases, and linked to interpretable state transitions.

### 9.2 What the framework enables, and what remains to be tested

The present article makes later empirical work more disciplined by specifying coding units, evidence spans, confidence fields, adjudication rules, variable-overlap handling, and prospective disconfirmation criteria. These elements do not replace inter-coder reliability, measurement validity, discriminant-validity assessment, state-space estimation, predictive performance, or causal explanation of the Iberian material. They make such work designable and open to critical inspection. The scientific claim is therefore precise: clinically meaningful collective-process variables can be specified in advance of validation in a form that is explicit enough to be tested, revised, or rejected.

### 9.3 Normative status and safeguards

The framework contains an unavoidable evaluative dimension at the clinical level, because terms such as tolerance, punitive reversal, progression, and destabilization are linked to suffering, functioning, and safety. At the collective level, however, this normativity must be transparent, functional, and methodological rather than civilizational or moralizing. The architecture does not claim that rigidity is always pathological or that plurality is always adaptive. It asks whether a regulatory configuration remains revisable, proportionate, evidence-sensitive, dignity-preserving, and capable of learning after threat has been contained.

This distinction matters because rigidity can sometimes be adaptive. Under acute threat, simplified procedure, strong boundaries, or rapid coordination may protect life and preserve basic order. The framework becomes clinically and politically useful only when it asks what such rigidity costs, how long it persists, whether it becomes moralized or genealogized, whether it suppresses alternative evidence, and whether revisability returns after the acute threat passes. PRO-C is therefore not treated as intrinsically “good,” and DEF-C/SUP-C are not treated as intrinsically “bad”; the relevant questions concern context, duration, proportionality, reversibility, harm, and institutional consequences.

### 9.4 Limitations and suitable uses

Several limitations define the appropriate use of the framework. First, it is a candidate architecture and not yet a validated measurement instrument. Second, the Iberian material is illustrative and cannot by itself test the architecture. Third, clinical process language can introduce anachronism if it is treated as a literal description of past actors rather than as a contemporary annotation vocabulary. Fourth, historical material is mediated by narrative, archival selection, translation, genre, and historiographic interpretation. Fifth, human-centered interpretation cannot abolish relativism; it can only make interpretive assumptions explicit, contestable, and subject to later reliability procedures. Sixth, comparative applications require temporal delimitation, because variables that are meaningful within one historical transition may not be comparable across distant periods without additional calibration.

These limitations do not negate the contribution. They specify the conditions under which a later pilot, comparative validation study, or human-in-the-loop modeling effort would become scientifically informative. The framework is most suitable for contexts in which rich textual, institutional, or historical material can be segmented into bounded units, coded under explicit evidence and uncertainty rules, and compared with alternative explanations. It is least suitable for automated scoring, retrospective diagnosis of cultures, decontextualized risk classification, or normative ranking of civilizations, religions, or political communities.

A further limitation is the visible dependence on the author’s prior representation program. PAD-S, CSA, the flat-affect reframing, and the PAD-S/CSA shared representation paper are directly relevant because this article extends that specific representation-first program (5–8). None of these prior papers is used as validation of the collective variables. To reduce dependency on self-citation, the manuscript anchors the extension in independent literatures on Active Inference, metaphor identification, observer-based psychotherapy process coding, V-Dem-style expert aggregation, event coding, threat-rigidity theory, organizational threat-response research, and political psychology (14–16, 27–35).

## 10 Conclusion

This article offers a clinically interpretable, human-centered candidate annotation architecture that connects psychotherapy process theory, Active Inference, moral metaphor research, and social psychoanalysis. Its central proposal is that collective regulation under stress can be described through a small set of operational companion variables: DEF-C, ANX-C, PRO-C, SUP-C, and FRAME-C. These variables are not diagnoses of societies, emic descriptions of historical actors, or independently validated measurement constructs; they are candidate annotation targets designed to make future reliability testing, discriminant-validity assessment, comparative coding, and formal modeling possible.

Early modern Iberia was used as a temporally delimited worked coding demonstration to show what the architecture may look like historically and why such an extension is analytically useful. The proposal remains modest in its validation claims but constructive in its methodological aim. Its intended value is not to replace historical or social-scientific judgment with a mechanistic scoring system, but to make clinically informed, human-centered interpretation more explicit, accountable, and prepared for future empirical testing. If later studies show that the variables can be coded reliably, distinguished from simpler explanations, and used across contrastive cases, the framework could support a genuinely human-centered pathway from interpretive clinical theory to responsible computational modeling.

## Data Availability

The original contributions presented in the study are included in the article/**Supplementary Material**. Further inquiries can be directed to the corresponding author.

## Supplementary material

Supplementary Material S1 Contains the extended crosswalk, general annotation architecture, specimen codebook, ordinal encoding scheme, state-space-compatible encoding options, illustrative micro-coding examples, and staged validation roadmap. These materials are provided as an annotation protocol for future pilot work and do not constitute completed empirical validation.
