# Supplementary material for: A theory-driven candidate annotation architecture for collective regulation under stress in human-centered computational psychiatry: early modern Iberia as a worked coding demonstration
Source: Front Psychiatry. 2026 May 26;17:1859236. doi: 10.3389/fpsyt.2026.1859236 (PMC13246629; doi:10.3389/fpsyt.2026.1859236)
Supplement: Supplementary Material S1 — Contains the extended crosswalk, general annotation architecture, specimen codebook, ordinal encoding scheme, state-space-compatible encoding options, illustrative micro-coding examples, and staged validation roadmap. These materials are provided as an annotation protocol for future pilot work and do not constitute completed empirical validation. [file DataSheet1.pdf]

Supplementary Material S1

Extended annotation protocol and coding tables

Purpose. This supplement contains the extended annotation apparatus for the manuscript “A Theory-Driven Candidate Annotation Architecture for Collective Regulation Under Stress in Human-Centered Computational Psychiatry: Early Modern Iberia as a Worked Coding Demonstration.” The main manuscript retains the compact variable overview, prospective disconfirmation criteria, and minimal validation roadmap; the tables below preserve the technical detail needed for pilot annotation, reliability testing, adjudication, and later human-in-the-loop computational modeling.

Status of the material. The tables are pre-validational. They make a later empirical pilot designable and open to critical inspection; they do not report inter-coder reliability, measurement validity, parameter estimation, or predictive performance.

Supplementary Table S1. Extended crosswalk from source traditions to the proposed collective-process framework

Tradition	Core construct	Contribution to the present model
PAD-S / CSA	DEF, ANX, PRO, SUP plus safety thresholds	Provides a clinically interpretable micro-process grammar that preserves load-bearing distinctions and keeps safety explicit.
Active Inference	Shared generative models, precision weighting, uncertainty reduction	Supplies a formal account of how actors stabilize prediction under threat and how socially shared models can become rigid, self-reinforcing, or exploratory.
Conceptual Metaphor Theory / Moral Politics	Nation-as-family; strict-father and nurturant-parent moral frames	Explains how moral and political meaning is encoded, transmitted, and made intuitively legible at scale.
Social psychoanalysis / social defense	Social character; institutionalized defense; group basic assumptions	Links collective meaning systems to recurring defenses, role expectations, and institutional responses to anxiety.
Adjacent threat-response literatures	Threat-rigidity, motivated social cognition, organizational threat response	Provides independent empirical and methodological anchors for the threat → rigidity → closure transition logic.

Supplementary Table S2. General annotation architecture for collective companion variables

Field	Proposed specification
Primary coding unit	Episode: the smallest coherent text unit with an interpretable claim about threat, norm, sanction, boundary drawing, revision, or moral framing. Depending on corpus, this may be a paragraph, decree article, protocol entry, accusation point, interrogation step, chronicle note, or event description.
Context unit	Full document or source item plus at least one preceding and one following unit; metadata include date, institution, genre, speaker/actor, and source type.
Temporal aggregation	For state models, coded units can be aggregated secondarily into time bins, such as document-internal sequence steps, months, years, or case-defined historical phases.
Coding format	Multi-label ordinal coding: DEF-C, ANX-C, PRO-C, and SUP-C use 0–3. FRAME-C is split into direction (−2 to +2) and rigidity (0–3).
Evidence requirement	Every code ≥1 requires at least one explicit evidence span or a clearly paraphrasable indicator in the coded unit.
Missing information	NA/unclear is distinct from 0. Code 0 means no sufficient evidence in the unit, not that the phenomenon is historically absent.
Uncertainty	Confidence is coded as 1 = low, 2 = medium, 3 = high and indexes evidence clarity plus codebook fit, not authorial certainty or historical truth. Low-confidence units remain usable for training and adjudication but should not anchor substantive claims alone.
Primary/secondary coding	When more than one function is supported by evidence spans, coders assign a primary code for the dominant immediate regulatory function and secondary codes for additional supported functions. Co-coding is allowed but must be evidence-linked.
Double coding	The pilot should be fully double-coded; in the main phase at least 15–20% overlap should be maintained, with drift checks.
Adjudication	Mandatory when coders differ by more than one ordinal level, when FRAME-C direction changes sign, or when coders disagree between DEF-C vs. SUP-C or PRO-C vs. SUP-C.

Supplementary Table S3. Specimen codebook for the five collective companion variables

Variable	Operational unit and indicators	Decision rule	Examples and adjudication
DEF-C	Text segment or institutional episode. Indicators: scapegoating, impurity/contamination semantics, reduction of complex causes to deviant groups, exclusion of ambiguous actors, ritualized boundary policing.	Code DEF-C only when disturbance or threat is attributed to an externalized or liminal object and this attribution reduces complexity. Ordinary boundary maintenance without externalization is insufficient.	Positive: disorder is attributed to a suspicious minority whose exclusion would “cleanse” the community. Negative: a report of crop failure and taxation problems without a deviant group being marked as cause. If sanction is central, co-code SUP-C.
ANX-C	Context unit, crisis text, event cluster, institutional communication. Indicators: emergency, invasion, contamination, breakdown, moral panic, repeated uncertainty markers, rejection of plural interpretations.	Code ANX-C when threat, uncontrollability, or ambiguity intolerance is explicit or strongly grounded in documented context. Do not infer ANX-C automatically from repression.	Positive: repeated statements that confusion and impurity endanger the body politic and require unification. Negative: purely administrative rule update without crisis language. ANX-C is a context variable and should not be back-coded from SUP-C.
PRO-C	Reform episode, negotiated settlement, inclusion practice, procedure for revision. Indicators: rule revision, reintegration, repair, explicit tolerance of ambiguity, plural conflict processing.	Code PRO-C when a unit organizes repair, revision, or inclusion as a legitimate solution. Tactical exception or uncontrolled fragmentation is insufficient.	Positive: a procedure allows disputed membership cases to be reviewed and reintegrated without humiliation. Negative: a temporary permit without rule change or inclusion logic. PRO-C may co-occur with ANX-C in transition phases.
SUP-C	Trial step, sanction, ritual, denunciation, public obedience test. Indicators: public humiliation, moral accusation, exemplary punishment, inherited taint, inquisitorial or quasi-inquisitorial procedure.	Code SUP-C when deviation is moralized and handled through shame, punishment, coercive correction, or obedience testing. Technical sanction without moral loading is insufficient.	Positive: deviation is treated as moral infection and processed through public examination and exemplary punishment. Negative: quiet administrative penalty without shame or moral accusation. If externalized group danger is central, co-code DEF-C.
FRAME-C	Speech, sermon, decree, law, manifesto, chronicle passage, repeated rhetorical pattern. Indicators: community-as-family, strict authority, purity, unity, obedience, or alternatively care, repair, protection, plurality.	Code two fields: direction (−2 care/plurality to +2 strict-order/purity) and rigidity (0–3). Code only when framing is repeated or structure-bearing, not for isolated moral phrases.	Positive: the community is repeatedly described as a threatened family requiring strict authority, purity, and obedience. Negative: isolated moral wording without governance function. Direction and rigidity are adjudicated separately.

Supplementary Table S4. Ordinal encoding scheme

Field	Values	Meaning
DEF-C / ANX-C / PRO-C / SUP-C	0	No sufficient evidence in the coded unit.
NA/unclear	NA	Insufficient source information, ambiguous unit boundary, or no interpretable evidence span. NA is not scored as 0 and should be excluded or modeled separately.
DEF-C / ANX-C / PRO-C / SUP-C	1	Isolated or weak indicator.
DEF-C / ANX-C / PRO-C / SUP-C	2	Repeated, salient, or functionally important indicator.
DEF-C / ANX-C / PRO-C / SUP-C	3	Dominant, structure-bearing, or institutionally enacted pattern.
FRAME-C direction	−2 to +2	−2 strongly care/plurality oriented; −1 moderately care/plurality oriented; 0 mixed/unclear; +1 moderately strict-order/purity oriented; +2 strongly strict-order/purity oriented.
FRAME-C rigidity	0 to 3	0 flexible/revisable; 1 mildly fixed; 2 clearly rigid; 3 strongly rigid/nearly unrevisable.
Confidence	1 to 3	1 low, 2 medium, 3 high confidence in evidence clarity and codebook fit; not confidence that the phenomenon is historically true beyond the coded evidence.

Supplementary Table S5. State-space-compatible encoding strategies

Strategy	Representation	Use case	Strength / limitation
Ordinal observation vectors	y_t = [DEF, ANX, PRO, SUP, FRdir, FRrig]	First pilot phase	Directly interpretable and reviewer-friendly; measurement error is only partly modeled.
Probabilistic aggregation	p(y_tj = k) rather than single value	Multiple coders or uncertain units	Keeps uncertainty visible; requires more reporting discipline.
Latent ordinal variables	Latent z_t observed through ordinal items	Several indicators per variable	Robust against single miscoding; higher modeling burden.
HMM / regime model	Discrete latent regimes	Sequences and transitions	Well suited for state changes; more model-driven.
Time-bin aggregation	Mean or distribution within period	Historical or institutional corpora	Practical for comparison; loses some microsequence information.
Span-based multi-label annotation	Evidence spans plus variable labels	Metaphor-rich or overlapping text markers	Source-sensitive; reliability is harder and may require unitized agreement.

Supplementary Table S6. Illustrative micro-coding examples for the internal Iberian contrast and brief Dutch Republic sketch

Source type / episode	Observable cue	Primary code	Why not another code? / caution
Public orthodoxy examination / denunciation procedure	Deviation is framed as moral infection and processed through public examination, confession pressure, or exemplary sanction.	SUP-C = 2–3; possible DEF-C co-code	SUP-C is primary if shame and punishment are the immediate regulatory function. DEF-C is secondary if the deviant actor is also externalized as boundary threat.
Blood-purity reasoning about conversos or Moriscos	Difference is described as inherited, transmissible, or genealogically suspicious even after formal conversion.	FRAME-C rigidity = 2–3; DEF-C = 2–3	This is not merely classification if ambiguity is converted into inherited danger. Avoid coding as SUP-C unless sanction or shame is explicit.
Administrative doctrinal clarification	The text specifies doctrine or jurisdiction without public shame, punishment, or externalized causality.	No SUP-C; possibly FRAME-C = 1	Strong religious content alone is insufficient for SUP-C or DEF-C. The regulatory function must be observable.
Layered coexistence under constraint in al-Andalus or mixed Iberian settings	Cross-confessional scholarship or local arrangement preserves interaction across difference through negotiated practice, multilingual exchange, or revisable procedure under asymmetric conditions.	PRO-C = 1–2; possible FRAME-C mixed	Do not romanticize coexistence. The row codes managed plurality under constraint, not egalitarian harmony or absence of domination.
Expulsion sequence framed by impurity or unrevisable inherited suspicion	Ambiguous membership is resolved by exclusion, and difference is made non-corrigible.	DEF-C = 3; FRAME-C rigidity = 3; possible SUP-C	The event may have economic or political causes as well. The code captures regulatory framing, not total historical causation.
Urban toleration arrangement in the Dutch Republic	Pragmatic coexistence is maintained through regulated plurality, compartmentalized worship, commercial mediation, or local compromise despite continued confessional tension.	PRO-C = 2; FRAME-C direction = −1/0; rigidity = 1	Useful as an external contrastive sketch because conflict and hierarchy remain present. Do not mistake pragmatic toleration for full liberal equality or absence of threat.

Supplementary Table S7. Extended staged validation roadmap

Stage	Goal	Concrete test	Minimum data / output
1. Conceptual and codebook validation	Ensure variables are intelligible, distinct, and not so broad as to become empty.	Expert review by clinical, historical, and computational readers; codebook revision log.	Manual v1.0 with examples, negative cases, and adjudication rules.
2. Reliability	Show that independent coders can use the variables consistently.	Weighted Krippendorff's alpha for ordinal variables; weighted Cohen's kappa for two coders; unitized agreement if spans vary [34,35].	Double-coded pilot of 80–120 units, then 15–20% overlap in main coding.
3. Construct validity	Show expected links with external or adjacent indicators.	ANX-C with documented threat proxies; SUP-C with sanction/denunciation records; FRAME-C with metaphor clusters; PRO-C with revision/inclusion procedures.	Coded corpus plus contextual markers.
4. Sequence / predictive validity	Test whether the proposed transition grammar has temporal structure.	Lagged ordinal models, transition matrices, HMM/regime models, or state-space models.	Sequenced episodes, time bins, and at least one internal/external contrast condition.
5. Model comparison	Assess added value over simpler labels.	Compare full model against ideology-only, event-count-only, sentiment-only, or punitive-only baselines.	Same corpus, same train/test or comparison design.
6. Practical utility	Assess whether the framework improves human interpretation.	Annotation time, coder feedback, interpretability ratings, supervision/education utility.	Human-in-the-loop workflow data.

Use with main manuscript. These supplementary tables are intended to be read together with Sections 5, 6, and 8 of the main manuscript. In any empirical implementation, the codebook should be revised after coder training and again before formal reliability estimation.
